# Ultra-Low-Loss Mid-Infrared Plasmonic Waveguides Based on Multilayer Graphene Metamaterials

**DOI:** 10.3390/nano11112981

**Published:** 2021-11-06

**Authors:** Chia-Chien Huang, Ruei-Jan Chang, Ching-Wen Cheng

**Affiliations:** 1 Institute of Nanoscience, National Chung Hsing University, Taichung 40227, Taiwan; 2 Department of Physics, National Chung Hsing University, Taichung 40227, Taiwan; grunt230@yahoo.com.tw (R.-J.C.); mikasa210549@gmail.com (C.-W.C.)

**Keywords:** graphene, multilayer, mid-infrared photonic, plasmonic waveguide, metamaterials, nano-optics, field enhancement

## Abstract

Manipulating optical signals in the mid-infrared (mid-IR) range is a highly desired task for applications in chemical sensing, thermal imaging, and subwavelength optical waveguiding. To guide highly confined mid-IR light in photonic chips, graphene-based plasmonics capable of breaking the optical diffraction limit offer a promising solution. However, the propagation lengths of these materials are, to date, limited to approximately 10 µm at the working frequency *f* = 20 THz. In this study, we proposed a waveguide structure consisting of multilayer graphene metamaterials (MLGMTs). The MLGMTs support the fundamental volume plasmon polariton mode by coupling plasmon polaritons at individual graphene sheets over a silicon nano-rib structure. Benefiting from the high conductivity of the MLGMTs, the guided mode shows ultralow loss compared with that of conventional graphene-based plasmonic waveguides at comparable mode sizes. The proposed design demonstrated propagation lengths of approximately 20 µm (four times the current limitations) at an extremely tight mode area of 10^−6^ *A*_0_, where *A*_0_ is the diffraction-limited mode area. The dependence of modal characteristics on geometry and material parameters are investigated in detail to identify optimal device performance. Moreover, fabrication imperfections are also addressed to evaluate the robustness of the proposed structure. Moreover, the crosstalk between two adjacent present waveguides is also investigated to demonstrate the high mode confinement to realize high-density on-chip devices. The present design offers a potential waveguiding approach for building tunable and large-area photonic integrated circuits.

## 1. Introduction

Significant enhancements achieved in light–matter interactions, nonlinear optical effects, chemical and biological sensing sensitivities, and resolution in imaging and spectroscopy can further benefit from the localization of light waves in deep subwavelength sizes. To date, the most promising approach to achieve this goal is to excite surface plasmon polaritons (SPPs) [1] by coupling photons and free electrons at metal–dielectric interfaces. For visible and near-infrared (IR) wavelengths, noble metals have been used to build various nanoscale photonic circuits [2,3,4,5]. However, alternative metallic materials are necessary because of the weak confinement of SPP modes in conventional noble metals for mid-IR and terahertz (THz) bands [6,7]. Graphene [8,9,10,11,12] is an emerging 2D material with extraordinary electric, thermal, and optical properties that can be flexibly tuned by electrical gating or chemical doping. Graphene is considered a promising candidate for SPP waveguiding in the mid-IR [6,7,9,11,12] and THz [9,12] ranges because of its nearly pure imaginary surface conductivity and extreme light confinement. The real and imaginary parts of the surface conductivity of graphene characterize the ohmic loss and magnitude of the wavevector, respectively. At the spectral bands of mid-IR and THz, the real part of graphene’s surface conductivity approaches zero, leading to a comparatively low ohmic loss. Because of their superior merits, many graphene-based optoelectronic and photonic devices, including polarizers [13,14,15,16], modulators [17,18,19,20,21], sensors [22,23,24], switches [25,26,27,28], and couplers [29], have been reported in recent years. To design functional optical devices well, detailed analyses of the mode properties of graphene plasmonic waveguides (GPWs) are of essential importance.

Many studies [30,31,32,33,34,35,36,37,38,39] have reported a variety of GPWs operating in the mid-IR range to improve SPP mode performance. For a plasmonic waveguide, several indices to evaluate the waveguiding performance of a device include the normalized mode area (*A_m_*); the propagation length (*L_p_* = *λ*/[4*π*Im(*n_e_*)]), where *λ* is the working wavelength in free space and Im(*n_e_*) is the imaginary part of the effective refractive index; and the figure of merit (*FoM*), which indicates the ratio between *L_p_* and mode size. For a dielectric loaded GPW, Xu et al. [31] theoretically investigated the dependence of the *L_p_* of fundamental graphene-based SPP (GSPP) modes on the geometry parameter, frequency (*f*), Fermi energy (*E_F_*), and carrier mobility (*µ*) of graphene. At *f* = 20 THz, they obtained *L_p_* < 3 µm at *E_F_* = 0.6 eV and *µ* = 1 m^2^/V·s (the same value is used for subsequent comparisons, unless stated otherwise). Note that the calculations of *L_p_* in Refs. [31,32,33,34,35,36,37,38,39] are unified by the same definition as mentioned above. Liu et al. [32] proposed a symmetrical long-range GSPP hybrid waveguide on a silica (SiO_2_) buffer layer on a silicon (Si) substrate showing an *Lp* of approximately 5 µm and an extremely confined area, *A_m_* = 8.0 × 10^−7^, at *f* = 30 THz and *E_F_* = 0.8 eV. Considering a pattern-free suspended graphene sheet over a Si ridge substrate, Bahadori-Haghighi et al. [33] numerically demonstrated a low-loss GPW with *L_p_* = 9 µm at *f* = 30 THz and *E_F_* = 0.35 eV.

In addition to these GPWs [31,32,33], researchers have focused on another kind of GPW, called graphene-coated nanowire waveguide (GCNW)-related structures [34,35,36,37,38,39]. Gao et al. [34,35] showed that a single GCNW performs low propagation loss and is cutoff free for the fundamental mode. At *f* = 30 THz, a single GCNW with a radius of *R* = 100 nm and permittivity *ε_NW_* = 2.1 shows *L_p_* = 4 µm and *A_m_* = 9.0 × 10^−4^ at *E_F_* = 0.6 eV for the fundamental mode. To improve the weak confinement of a radically polarized mode in a single GCNW [34,35], Teng et al. [36] adopted a GCNW dimer to couple two GCNW modes. The mode properties were moderately improved to *L_p_* = 4 µm and *A_m_* = 9.0 × 10^−5^ at *E_F_* = 0.6 eV and *f* = 30 THz. To significantly enhance the mode localization, Liu et al. [37] reported a two-layer dielectric GCNW composed of a Si core surrounded by a SiO_2_ layer and an outermost graphene sheet, forming a conventional hybrid plasmonic waveguide (HPW) structure [40]. They obtained an *A_m_* of approximately 1.5 × 10^−5^ and an *L_p_* of approximately 1.5 µm at *E_F_* = 0.5 eV operating at a wavelength of *λ* = 7 µm (about *f* = 42.85 THz). The cost for improving energy confinement is reflected in a moderately shorter *L_p_*. Later, Liu et al. [38] extended their design by adding an extra graphene sheet between the Si and SiO_2_ layers to form a symmetric long-range coupling mode with an *L_p_* of ~10 µm and *A_m_* of ~10^−5^ at *E_F_* = 0.6 eV and *f* = 30 THz. Last year, Teng et al. [39] adopted a GCNW-loaded Si nano-rib (GCNWLSNR) structure based on the coupling between the GCNW plasmon mode and the Si nano-rib to significantly shrink the mode size to *A_m_* = 9.8 × 10^−7^ while keeping *L_p_* at ~9 µm at *E_F_* = 0.6 eV and *f* = 20 THz. The experimental fabrication approach to a GCNW, by rolling a graphene ribbon, was presented in refs. [41,42] to demonstrate practical feasibility.

In this study, we propose a high-performance GPW based on multilayer graphene metamaterials (MLGMTs) [43,44] on a Si nano-rib waveguide partially covered by a low-index porous SiO_2_ film. The fundamental volume plasmon polariton (VPP) modes, which are supported by MLGMT-coupling SPPs at individual graphene sheets [45], are coupled with a dielectric mode supported by a Si nano-rib waveguide. The resulting coupled mode performs not only deep subwavelength mode confinement but also ultra-low loss through introducing the high-conductivity MLGMTs compared with those of previously published results [32,33,34,35,36,37,38,39]. The proposed approach provides an additional degree of freedom, the number of graphene layers, to control the mode characteristics. Moreover, fabrication imperfections and spectral response are also addressed to evaluate the robustness and operating bandwidth of the proposed structure.

## 2. Waveguide Structure and Methods

**Figure 1:**
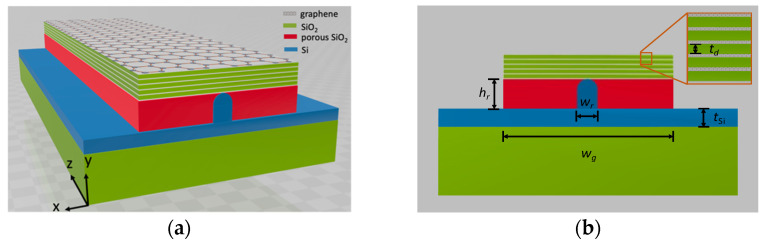
Schematic of the proposed waveguide structure—(**a**) 3D structure and (**b**) cross section of (**a**) with a zoomed-in view of the MLGMTs.

A 3D schematic diagram of the GPW and its front view are shown in Figure 1a,b, respectively. The structure consists of a porous SiO_2_ [46,47] layer sandwiched by MLGMTs, which are formed by alternating graphene–dielectric layers, and a Si nano-rib waveguide deposited on a conventional SiO_2_ substrate. Here, the same SiO_2_ as the substrate is chosen as the dielectric layers in the MLGMTs. Following the HPW structure [40], the MLGMTs, porous SiO_2_, and Si nano-rib are considered the metal, low-index dielectric, and high-index dielectric layers, respectively. Therefore, the mode field of the present structure can be strongly squeezed in the nanoscale region between the Si nano-rib and the MLGMTs, thus achieving ultrasmall mode sizes.

**Figure 2:**
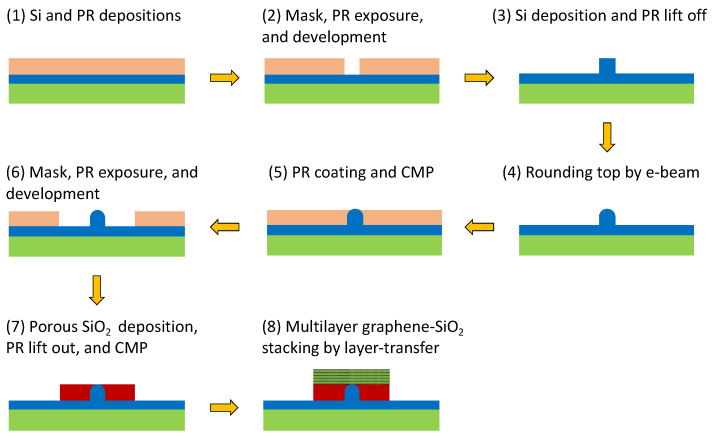
Schematic of the fabrication process for the proposed waveguide structure.

The fabrication steps are schematically shown in Figure 2 and are described as follows:

(1) Si and positive photoresist (PR) films with thickness *h_r_* (nm) are deposited on a conventional SiO_2_ substrate; (2) a mask is applied and followed by PR exposure and PR development to define a rectangular groove; (3) a Si layer is deposited and followed by the lift off of the PR to form a Si nano-rib waveguide; (4) the top of the Si nano-rib is rounded using e-beam lithography by carefully controlling the exposure time and scanning speed; (5) a PR film is coated on the surface and is used to flatten the PR film using chemical mechanical polishing (CMP); (6) a mask is applied and followed by PR exposure and PR development to lift out the PR film and define the porous SiO_2_ region; (7) a porous SiO_2_ film is evaporated on the surface using the oblique deposition technique [46,47], the PR film is lifted off, and then CMP is used to flatten the porous SiO_2_ layer; finally, (8) forming multilayer graphene–SiO_2_ stacking by a chemical vapor deposition (CVD), a layer transfer method [48,49,50], or transfer-free, solution-phase deposition method [51,52]. Considering the high-technique step to form the MLGMTs, we describe the main fabrication processes as follows. The former approaches [48,49,50] include depositing graphene on a copper foil, transferring it to the substrate using the PMMA transfer technique, etching the copper foil using ammonium persulfate, doping the graphene by soaking the sample in a suitable solution, depositing the target dielectric layer by the atomic layer deposition being able to carefully control its thickness, and finally repeating the processes to achieve the desired number of layers. Note that the number of graphene layers in [50] can reach 11 layers. For the latter approach [51,52], the fabrication processes include the use of graphene-oxide (GO) in solution, deposition directly on a dielectric layer without requiring a transfer process, repetition to achieve the desired number of GO layers, and finally laser photoreduction from GO to graphene with removing the oxygen-functional groups, in which the bandgap of the graphene can be tuned by varying the lase power. Note that the number of graphene layers in [52] can reach 20 layers. Compared to the CVD transfer method [48,49,50], the advantages of the solution-phase method [51,52] are transfer-free, with quality independent of the number of layers, and controllable for the optical responses by tunning the bandgap of graphene. Consequently, the fabrication of the multilayer graphene metamaterials may be not that challenging and have complex techniques required and is moderately achievable using modern fabrication techniques, making the proposed waveguide structure practically realizable. Note that the conventional electrical gating on graphene layers uses a single voltage to a top contact [53,54], thus resulting in an inhomogeneous chemical potential of the graphene layers varying from layer to layer due to interlayer screening [55] in a multilayer graphene structure. The non-uniformity of chemical potential is more significant as the number of graphene layers increases. As a result, a potential scheme [49] can be adopted to achieve the required chemical potential in the proposed MLGHPW. This approach controls the Fermi energy levels of individual graphene layers by different gate voltages, making the carrier concentrations alter together in all layers.

In theory, the MLGMTs can be considered a coupled system with interacting multiple graphene sheets supporting multiple nondegenerate plasmon modes (called VPP modes). The VPP modes show hyperbolic isofrequency contours [45] and a large density of electromagnetic states, resulting in high-*k* guided modes. Among the VPP modes, the fundamental VPP mode (VPP_0_) shows the lowest loss, although it possesses a comparably larger mode size. By coupling the VPP_0_ with the dielectric mode of the Si nano-rib waveguide, a hybrid mode not only preserves the low-loss property of the VPP_0_, but also significantly benefits from the high mode confinement of the Si nano-rib. In the present structure, the geometry parameters are as follows: *w_g_* and *t_d_* are the width and thickness, respectively, of the dielectric (SiO_2_) layer; *N* is the number of MLGMT layers between graphene sheets (*N* + 1 layers); and *w_r_*, *h_r_*, and *t_Si_* are the width, height, and bottom thickness of the Si nano-rib waveguide, respectively. Here, we set the radius of curvature to *r* = *w_r_*/2. The relative permittivities of Si, conventional SiO_2_, and porous SiO_2_ at *f* = 20 THz are *ε_Si_* = 12.25 [56], *ε_SiO__2_* = 2.25 [56], and *ε_p-__SiO__2_* = 1.10 [46], respectively. Here, graphene is modeled as an infinitely thin sheet with a surface current density of *J* = *σE* in-plane, where *E* is the electric field vector and *σ* is the total surface conductivity of graphene with *σ* = *σ_intra_* + *σ_inter_*, including intraband (*σ_intra_*) and interband (*σ_inter_*) contributions, which can be calculated using the local random phase approximation [57]:

(1) $$\sigma_{\mathrm{int}ra}(\omega ,E_{F},\tau ,T)=\frac{-j2e^{2}k_{B}T}{\pi \hbar^{2}(\omega -j\tau^{-1})}\ln\left[2\cosh\left(\frac{E_{F}}{2k_{B}T}\right)\right]$$

and

(2) $$\sigma_{\mathrm{int}er}(\omega ,E_{F},\tau ,T)=\frac{e^{2}}{4\hbar }\ln\left[\begin{array}{l} \frac{1}{2}+\frac{1}{\pi }\tan^{-1}\left(\frac{\hbar \omega -2E_{F}}{2k_{B}T}\right)+\\ \frac{j}{2\pi }\ln\left(\frac{{\left(\hbar \omega +2E_{F}\right)}^{2}}{{\left(\hbar \omega -2E_{F}\right)}^{2}+{\left(2k_{B}T\right)}^{2}}\right) \end{array}\right],$$

where *E_F_* is the Fermi energy, *τ* = *µE_F_/eV_F_*^2^ is the carrier relaxation lifetime, *T* is the temperature, *k_B_* is the Boltzmann constant, ℏ is the reduced Planck constant, *e* is the electron charge, *µ* is the carrier mobility in graphene, and *V_F_* = 10^6^ m/s is the Fermi velocity of electrons. Here, we consider a carrier mobility, *µ* = 1 m^2^/V·s, at *T* = 300 K, which is also used to study the performance of GPWs [34,35,36,37,38,39]. To evaluate the waveguiding performance of plasmonic waveguides, the propagation length (*L_p_*), *FoM* ($L_{p}/2\sqrt{A_{m}/\pi }$ [40]), and normalized mode area (*A_m_* = *A_e_*/*A*_0_) are used, where *A*_0_ = *λ*^2^/4 (*λ* is the working wavelength) is the diffraction-limited mode area and *A_e_* is the effective mode area given by

(3) $$A_{e}=\frac{W_{m}}{W{\left(r\right)}_{\max}}=\frac{1}{W{\left(r\right)}_{\max}}\int_{-\infty }^{\infty }\int_{-\infty }^{\infty }\;W(r)\;dA\;.$$

Equation (3) includes the ratio of the total mode energy, *W_m_*, and the peak of the energy density, *W*(*r*), which is given by:

(4) W(r)=12{Re[dε(r)ωdω]|Ε(r)| 2+μ0|H(r)| 2},

where *ω* is the angular frequency, *ε*(*r*) is the profile of relative permittivity, *µ*_0_ is the permeability in a vacuum, and |**E**| and |**H**| are the intensities of the electric and magnetic fields, respectively. The numerical results are calculated using the COMSOL Multiphysics software based on the rigorous finite element method.

## 3. Results and Discussion

### 3.1. Waveguiding Performance Dependence on the Number of MLGMT Graphene Layers

**Figure 3:**
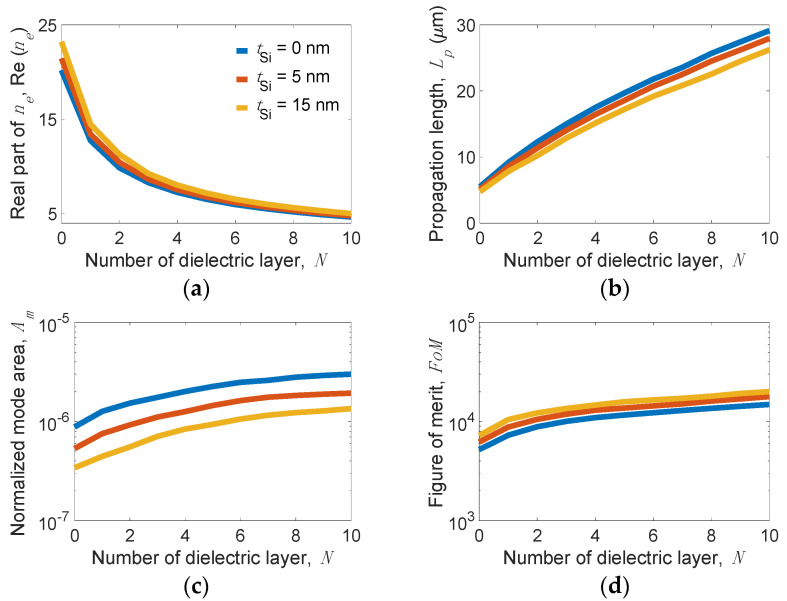
(**a**) Real part of effective index *n_e_*, Re(*n_e_*), (**b**) normalized mode area, *A_m_*, (**c**) propagation length, *L_p_*, and (**d**) figure of merit, *FoM* versus the number of dielectric layer, *N* for the present structure operating at *f* = 20 THz for different *t_Si_* values at parameters of *w_g_* = 200 nm, *t_d_* = 5 nm, *w_r_* = 10 nm, *h_r_* = 30 nm, and *E_F_* = 0.6 eV.

Figure 3 shows the mode properties of the present structure versus *N* for several values of *t_Si_* at the following parameters: *w_g_* = 200 nm, *t_d_* = 5 nm, *w_r_* = 10 nm, *h_r_* = 30 nm, *E_F_* = 0.6 eV, and *f* = 20 THz. We observe that the real part of *n_e_*, Re(*n_e_*) sharply decreases from approximately 20 to 5 as *N* increases (see Figure 3a), but *A_m_* moderately increases from 5.34 × 10^−7^ to 1.93 × 10^−6^ for *t_Si_* = 5 nm (see Figure 3b). Note that the slopes of Re(*n_e_*) and *A_m_* versus *N* become smaller when *N* is greater than five, meaning that Re(*n_e_*) and *A_m_* are slightly influenced by larger *N* values. In contrast, *L_p_* linearly increases from 5.1 to 27.9 µm for *t_Si_* = 5 nm as *N* increases from 0 to 10 (see Figure 3c). Further increasing *L_p_* is only limited by the ability to fabricate an increasing number of *N*. The *FoM* increases from 6214 at *N* = 0 to 17,830 at *N* = 10 for *t_Si_* = 5 nm (see Figure 3d).

**Figure 4:**
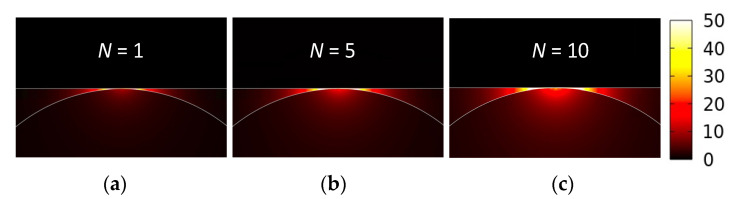
Mode fields of |**E**| for *N* = (**a**) 1, (**b**) 5, and (**c**) 10 at the same parameters as in Figure 3.

The obtained results show that the proposed design can achieve both an ultralong *L_p_* (up to ~28 µm) and an extremely small *A_m_* (on the order of 10^−6^). Comparing these values with *A_m_* = 9.8 × 10^−7^ and *L_p_* = 3.5 µm of the GCNWLSNR [39], the present structure improves *L_p_* by more than three times while maintaining a comparable order of magnitude of *A_m_*. For observing the mode profiles, Figure 4a–c depict the |**E**| values near the nanoscale region between the MLGMTs and Si nano-rib for *N* = 1, 5, and 10 at *t_Si_* = 5 nm. Clearly, increasing *N* results in a stronger peak of |**E**| but looser mode confinement, leading to the moderate increase in *A_m_* (see Figure 3b).

**Figure 5:**
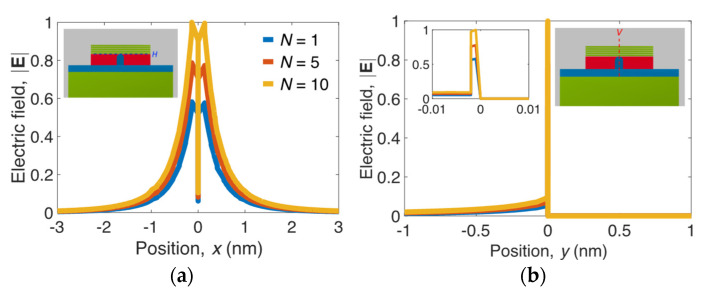
Electric fields, |**E**| along the (**a**) horizontal dashed line *H* and (**b**) vertical dashed line *V* shown in the corresponding insets for *N* = 1, 5, and 10.

Figure 5a,b also show 1D field plots along the horizontal dashed line *H* (inset of Figure 5a) and vertical dashed line *V* (inset of Figure 5b), respectively, to more clearly observe the relative mode profiles for different *N* values. The ultrasmall *A_m_* = 10^−6^ of the present design shows full widths at half maximums of approximately 1 and 0.1 nm along the *x* and *y* directions, respectively. The *L_p_* can be attributed to the mode profile being significantly shifted from the region of MLGMTs to the Si nano-rib as *N* increases (see Figure 5b). Note that the conditions *E_F_* = 0.6 eV, *N* = 10, and *f* = 20 THz are used in subsequent analyses unless stated otherwise.

**Figure 6:**
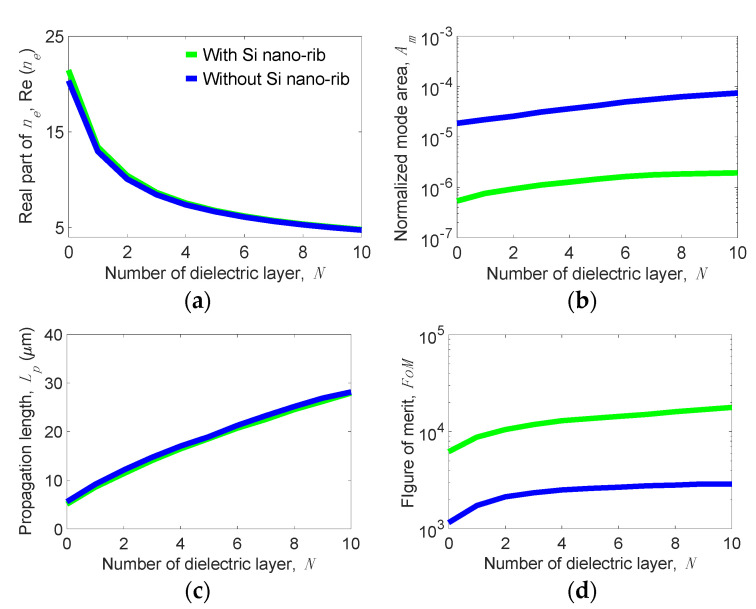
(**a**) Real part of effective index *n_e_*, Re(*n_e_*), (**b**) normalized mode area, *A_m_*, (**c**) propagation length, *L_p_*, and (**d**) figure of merit, *FoM* versus the number of dielectric layer *N* for the present structure with and without the Si nano-rib.

To clearly elucidate how the dielectric Si nano-rib changes the VPP_0_ mode, we analyze the properties of the VPP_0_ mode supported by the proposed structure without the nano-rib. Figure 6 shows the mode properties of the present structure with and without the nano-rib versus *N* at the same parameters as used in Figure 3 with *t_Si_* = 5 nm.

**Figure 7:**
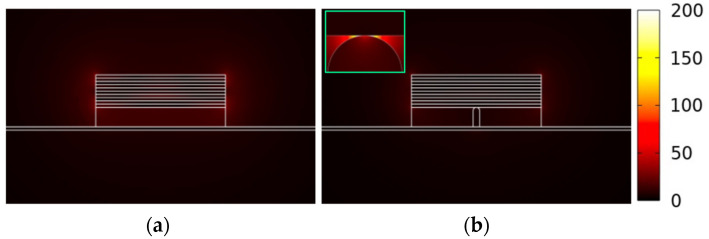
Mode fields of |**E**| for the present waveguide structure (**a**) without and (**b**) with the Si nano-rib.

We observe that Re(*n_e_*) and *L_p_* of the two structures are really close, but *A_m_* of the structure without the nano-rib is one to two orders of magnitude larger than that of the present structure as shown in Figure 6b, making the *FoM* of the structure without the nano-rib about one order of magnitude smaller. The above result reveals that the nano-rib can make the field distribution of the VPP_0_ mode significantly concentrated around the nanoscale region between the nano-rib and the MLGMTs. For observing the effect, Figure 7a,b show the |**E**| distributions of the present structure with and without the nano-rib, respectively, along with the zoomed-in view (see inset of Figure 7b) of the |**E**| distribution around the nano-rib. Without the nano-rib, the field profile spreads stronger out of the MLGMTs than that of the present design. In addition, the field of the present structure is significantly enhanced and focused mainly around the nano-rib, effectively shrinking its *A_m_*.

### 3.2. Mode Characteristic Dependence on Geometric Parameters

**Figure 8:**
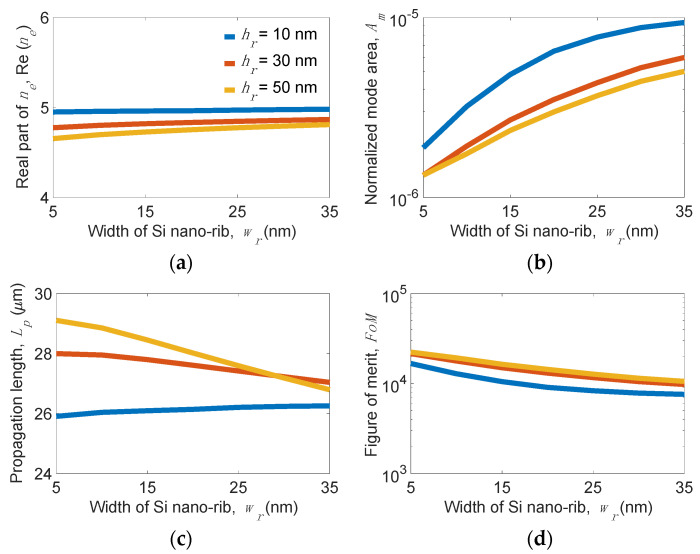
(**a**) Real part of effective index *n_e_*_,_ Re(*n_e_*), (**b**) normalized mode area, *A_m_*, (**c**) propagation length, *L_p_*, and (**d**) figure of merit, *FoM* of the present structure versus the width of Si nano-rib, *w_r_* for different height of Si nano-rib, *h_r_* values at the width of MLGMTs, *w_g_* = 200 nm, thickness of the bottom Si layer, *t_Si_* = 5 nm, and thickness of dielectric layers of MLGMTs, *t_d_* = 5 nm.

To fully assess the waveguiding performance of the proposed structure, we analyze the geometrical dependence of the mode properties. Figure 8 shows the mode properties versus *w_r_* for several *h_r_* values at *w_g_* = 200 nm, *t_Si_* = 5 nm, and *t_d_* = 5 nm. We observe that Re(*n_e_*) slightly depends on *w_r_* and *h_r_* (see Figure 8a) but *A_m_* shows a substantial dependence on *w_r_* and *h_r_* (see Figure 8b). For example, *A_m_* varies from 1.6 × 10^−6^ to 6.0 × 10^−6^, while *w_r_* changes from 5 to 35 nm at *h_r_* = 30 nm. This effect can be attributed to a larger *w_r_,* obviously leading to a looser mode confinement. On the other hand, larger *h_r_* attains tighter *A_m_* due to the increase in the area of a low-index region, making the mode field more concentrated towards the region between the MLGMTs and the Si nano-rib. However, *L_p_* significantly increases as *h_r_* increases, although *A_m_* shrinks. This is because the mode field shifts toward the Si nano-rib side as *h_r_* increases. Interestingly, *L_p_* slightly increases for the smaller *h_r_* = 10 nm but moderately decreases for the larger *h_r_* > 30 nm as *w_r_* increases (see Figure 8c). For example, *L_p_* varies from 29.2 (25.8) at *w_r_* = 5 nm to 26.8 (26.2) µm at *w_r_* = 35 nm for *h_r_* = 50 (10) nm. Note that smaller *w_r_* or larger *h_r_* (>20 nm here) can effectively improve the *FoM* of the proposed waveguide structure (see Figure 8d).

**Figure 9:**
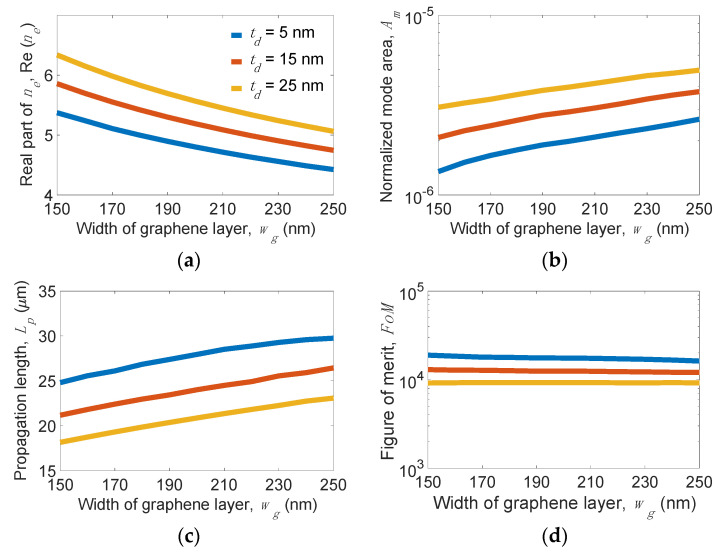
(**a**) Real part of effective index *n_e_*_,_ Re(*n_e_*), (**b**) normalized mode area, *A_m_*, (**c**) propagation length, *L_p_*, and (**d**) figure of merit, *FoM,* of the present structure versus *w_g_* for different *t_d_* values at *w_r_* = 10 nm, *h_r_* = 30 nm, and *t_Si_* = 5 nm.

Next, we consider the effects of *w_g_* and *t_d_* of the MLGMTs on mode properties. At *w_r_* = 10 nm, *h_r_*= 30 nm, and *t_Si_*= 5 nm, Figure 9 shows the mode properties versus *w_g_* for several *t_d_* values. We observe that *Re*(*n_e_*), *A_m_*, and *L_p_* moderately depend on *w_g_* and *t_d_*. We also observe that *A_m_* and *L_p_* decrease as *w_g_* decreases. Differing from increasing *h_r_*, which leads to a smaller *A_m_* (see Figure 8b), increasing *w_g_* increases *A_m_*, although they all increase the low-index region. This is because increasing *w_g_* also increases the width of the MLGMTs, leading to a looser mode field. The compensation between *A_m_* and *L_p_* makes the *FoM* almost constant for different values of *w_g_*. As for the *t_d_*, the smaller value leads to stronger coupling between SPP modes at individual graphene sheets. Therefore, smaller *t_d_* concurrently achieves lower loss and smaller mode size, breaking the trade-off between *A_m_* and *L_p_* to effectively improve the waveguiding performance of the proposed structure.

### 3.3. Fabrication Tolerance, Material Parameters of Graphene, and Spectral Response

**Figure 10:**
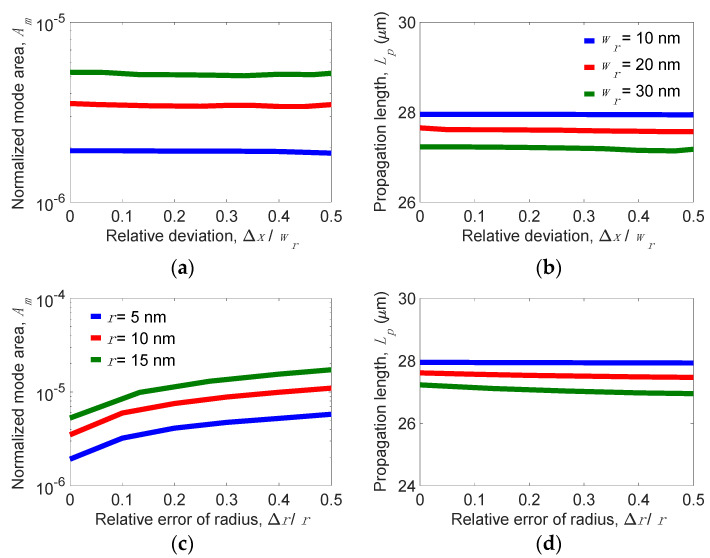
Dynamics of (**a**) normalized mode area, *A_m_,* and (**b**) propagation length, *L_p_,* versus the relative fabrication error of the width of the Si nano-rib (Δ*x*/*w_r_*) for different *w_r_* values; (**c**) *A_m_* and (**d**) *L_p_* versus the relative fabrication error of the radius of the curvature of the Si nano-rib (Δ*r*/*r*) for different *r* values when *h_r_* = 30 nm, *t_Si_* = 5 nm, *t_d_* = 5 nm, and *w_g_* = 200 nm.

In experiments, fabrication imperfections lead to a reduction in waveguiding performance. Among the geometric parameters, the strictest part of the structure that should be precisely fabricated is the dimension of the Si nano-rib. Figure 10a,b show the dependence of *A_m_* and *L_p_* on the relative fabrication error, Δ*x*/*w_r_*, at *h_r_* = 30 nm, *t_Si_* = 5 nm, *t_d_* = 5 nm, and *w_g_* = 200 nm, where Δ*x* is the fabrication error. Evidently, *A_m_* and *L_p_* are almost invariant in the range between Δ*x*/*w_r_* = 0 and 0.5. Similarly, the dependence of *A_m_* and *L_p_* on the relative fabrication error Δ*r*/*r*, where Δ*r* is the fabrication error, are shown in Figure 10c,d, respectively. At *r* = 10 nm, *A_m_* moderately varies from 4.2 × 10^−6^ to 8.5 × 10^−6^ while Δ*r*/*r* deviates from 0 to 0.5, but *L_p_* remains constant, although Δ*r*/*r* = 0.5. The analyzed results verify that the proposed structure possesses high fabrication tolerance on mode properties.

**Figure 11:**
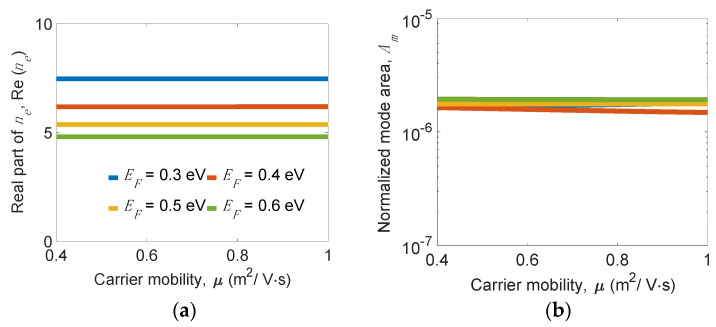

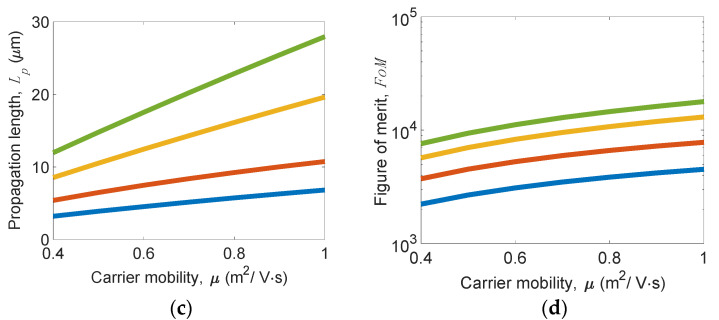
Plots of (**a**) real part of effective index *n_e_*_,_ Re(*n_e_*), (**b**) normalized mode area, *A_m_*, (**c**) propagation length, *L_p_*, and (**d**) figure of merit, *FoM,* of the present structure versus carrier mobility, *µ,* for several *E_F_*’s at *w_g_* = 200 nm, *w_r_* = 10 nm, *h_r_* = 30 nm, *t_Si_* = 5 nm, *t_d_* = 5 nm, and *f* = 20 THz.

Considering a high doping level of graphene that leads to reducing the carrier mobility (*µ*), we investigated the mode properties versus *µ* for several *E_F_*’s, as shown in Figure 11. We observe that Re(*n_e_*) and *A_m_* are nearly independent on *µ*; however, *L_p_* linearly reduces as *µ* decreases due to increasing the ohmic loss significantly for a higher doping level of *E_F_* > 0.5 eV. At *E_F_* = 0.4 eV, *L_p_* varies from 10.7 to 7.4 µm at *µ* = 1 and 0.6 (m^2^/V·s), respectively. On the other hand, a lower *E_F_* attains a moderately higher Re(*n_e_*) and slightly smaller *A_m_* showing a tighter mode confinement, but significantly leads to a shorter *L_p_* (see Figure 11c). For example, *L_p_* varies from 19.5 to 10.8 µm at *E_F_* = 0.5 and 0.4 eV, respectively, for the condition *µ* = 1 (m^2^/V·s). The results reveal that a lower *E_F_* or *µ* leads to a reduction in *L_p_* for general GPWs, mainly due to the higher ohmic losses.

**Figure 12:**
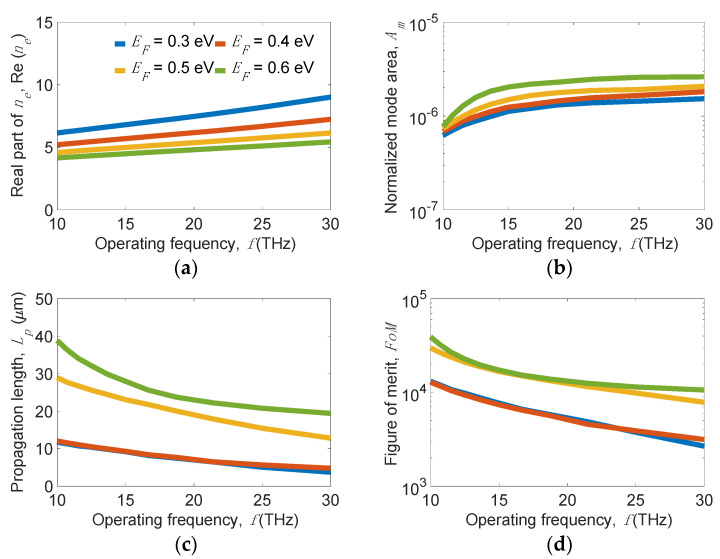
Plots of (**a**) real part of effective index *n_e_*_,_ Re(*n_e_*), (**b**) normalized mode area, *A_m_*, (**c**) propagation length, *L_p_*, and (**d**) figure of merit, *FoM,* of the present structure versus the operating frequency, *f*, for several values of *E_F_* when *w_g_* = 200 nm, *w_r_* = 10 nm, *h_r_* = 30 nm, *t_Si_* = 5 nm, and *t_d_* = 5 nm.

To fully study the tunability of graphene within a bandwidth range, we address the spectral response of mode properties. At *w_g_* = 200 nm, *w_r_* = 10 nm, *h_r_* = 30 nm, *t_Si_* = 5 nm, and *t_d_* = 5 nm, the results for several values of *E_F_* are shown in Figure 12. As the working frequency, *f*, increases from 10 to 30 THz, Re(*n_e_*) and *A_m_* moderately increase, but *L_p_* significantly decreases. Considering the effect of *E_F_*, Re(*n_e_*) increases, but *A_m_* and *L_p_* decrease because of the enhanced mode localization as *E_F_* decreases. Note that *A_m_* is slightly influenced while *L_p_* is significantly influenced by varying *E_F_*. At *f* = 10 (30) THz, our design achieves unprecedented long *L_p_* values of 12.1 (4.8) and 38.9 (19.4) µm while maintaining ultrasmall *A_m_* values of 6.8 × 10^−7^ (1.8 × 10^−6^) and 7.8 × 10^−7^ (2.6 × 10^−6^) for *E_F_* = 0.4 and 0.6 eV, respectively. Exceptionally, the obtained *FoM* values are higher than 10^4^ (3.0 × 10^3^) within *f* = 10–30 THz, even while operating at *E_F_* = 0.6 (0.3) eV.

The conventional electrical gating on graphene layers uses a single voltage to a top contact [50,51], thus resulting in an inhomogeneous chemical potential of the graphene layers varying from layer to layer due to interlayer screening [52] in a multilayer graphene structure. The non-uniformity of chemical potential is more significant as the number of graphene layers increases. As a result, a potential scheme [49] can be adopted to achieve the required chemical potential in the proposed MLGHPW. This approach controls the Fermi energy levels of individual graphene layers by different gate voltages, making the carrier concentrations alter together in all layers.

### 3.4. Waveguide Crosstalk

**Figure 13:**
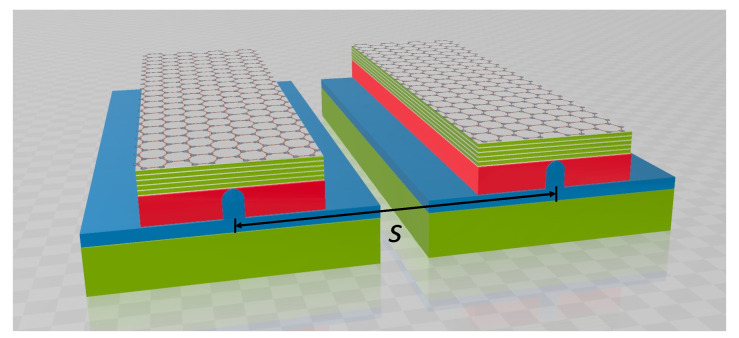
Schematic of a coupled waveguide consisting of two parallel MLGHPWs with a center-to-center separation *s*.

In addition to the size of *A_m_*, the crosstalk of the modes in adjacent waveguides complements to describe the degree of mode confinement and examines the feasibility for high integration of photonic integrated circuits. Figure 13 shows a coupled waveguide consisting of two parallel waveguides with a center-to-center separation *s*.

**Figure 14:**
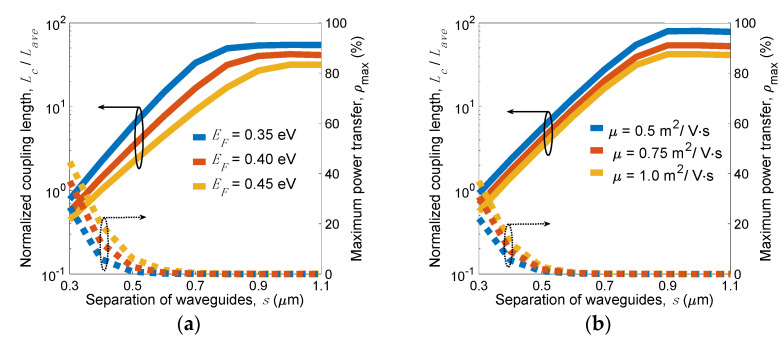
Normalized coupling length, *L_c_*/*L_ave_* and maximum transfer power, *ρ*_max_ as functions of separation of waveguides, *s* for several values of (**a**) *E_F_* at *µ* = 1 m^2^/V·s and (**b**) *µ* at *E_F_* = 0.4 eV for *f* = 30 THz and the geometry parameters *w_g_* = 180 nm, *w_r_* = 20 nm, *h_r_* = 30 nm, *t_Si_* = 25 nm, and *t_d_* = 25 nm.

According to the coupled mode theory (CMT) [58], energy exchange between the adjacent waveguides is due to the field coupling of evanescent tails of two normal modes. To evaluate the coupling strength, the coupling length of a lossless coupled waveguide system *L_c_* = *λ*/[*2*(*n_s_* − *n_a_*)] determines the length required to completely transfer power from one waveguide to another, where *n_s_* and *n_a_* are the *n_e_* of the symmetric and asymmetric modes, respectively. For a lossy waveguide system such as plasmonic waveguides, we adopted a more suitable criterion, the normalized coupling length *L_c_*/*L_ave_* [59], which considers both the power attenuation and maximum power transfer to measure the crosstalk, where *L_ave_* is the average *L_p_* of the symmetric and asymmetric modes. The maximum transfer power *ρ*_max_ between waveguides is only a function of *L_c_*/*L_ave_*, and the adjacent waveguides can be considered as nearly isolated (*ρ*_max_ = 0.33%) if *L_c_*/*L_ave_* > 10 is reached. This is because the transfer power from one channel to the other is relatively weak at the distance of *L_ave_*. At the frequency *f* = 30 THz and the geometry parameters of *w_g_* = 180 nm, *w_r_* = 20 nm, *h_r_* = 30 nm, *t_Si_* = 25 nm, and *t_d_* = 25 nm, the results of *L_c_*/*L_ave_* along with *ρ*_max_ versus *s* at *µ* = 1 m^2^/V.s for several *E_F_*s are shown in Figure 14a and those at *E_F_* = 0.4 eV are shown in Figure 14b for several *µ*s. The results show that decreasing *E_F_* or *µ* leads to weaker coupling strength, and the dependence of coupling strength on *E_F_* is stronger than that on *µ*. We observe that the separations for negligible couplings between waveguides are *s* = 0.52, 0.64, and 0.72 µm for *E_F_* = 0.35, 0.40, and 0.45 eV, respectively (see Figure 14a), and are *s* = 0.57, 0.61, and 0.64 µm for *µ* = 0.5, 0.75, and 1 m^2^/V.s, respectively (see Figure 14b). The results demonstrated that the coupling strength can be tuned by varying *E_F_* or *µ*, respectively. For instance, *ρ*_max_ achieves about 20% at *E_F_* = 0.45 eV and 5% at *E_F_* = 0.35 eV for the condition *µ* = 1 m^2^/V.s and *s* = 0.4 µm. The small values of *s* (about 0.5 to 0.7 µm) make the proposed waveguide capable of building high-density graphene-based photonic-integrated circuits operating in the mid-infrared range.

### 3.5. Comparison of Waveguiding Performance

To demonstrate the superior waveguiding performance of the proposed design, we compared the reported results [32,34,36,38,39] in Table 1 with our mode properties at the following parameters: *w_g_* = 200 nm, *w_r_* = 10 nm, *h_r_* = 30 nm, *t_Si_* = 5 nm, *t_d_* = 5 nm, *E_F_* = 0.6 eV, and *N* = 10. All the results in Table 1 were calculated at *f* = 30 THz, *µ* = 1 m^2^/V·s, and *E_F_* = 0.6 eV, except the results in ref. [32] with *E_F_* = 0.8 eV. If *E_F_* = 0.8 eV is decreased to 0.6 eV, *A_m_* and *L_p_* will be further reduced. Liu et al. [32] achieved an extremely small area, *A_m_* = 8.0 × 10^−7^, by adopting a symmetrical HPW. Teng et al. [36] proposed a GCNW dimer to cause the coupling of the fundamental modes between two GCNWs to improve the *A_m_* of the single GCNW [34] by one order of magnitude while keeping the same *L_p_*. Liu et al. [38] extended their previous report [32] to add an extra graphene sheet between Si and SiO_2_ layers and roll it into a cylindrical waveguide. It can be inferred that the two-layer graphene structure effectively improves the *L_p_* but at the cost of a larger *A_m_*. Last year, Teng et al. [39] proposed a GCNWLSNR structure composed of a single GCNW deposited on a Si nano-rib, which showed a performance of *L_p_* = 3.5 µm and *A_m_* = 2.0 × 10^−6^. In comparison with these published results [32,34,36,38,39], the proposed structure achieves an unprecedented waveguiding performance of *L_p_* = 19.4 µm and *A_m_* = 2.6 × 10^−6^, thus obtaining an extremely high value of *FoM* = 10,612.

Currently, the obstacles for experimentally fabricating integrated graphene waveguides include three major points, despite the mature developments of both silicon photonics and graphene industries. (1) Excitation of extremely high-*k* SPP modes supported by monolayer graphene; (2) high-efficiency coupling between the nanoscale mode sizes of high-*k* SPP modes and submicron-scale dielectric waveguide modes or micron-scale optical fiber modes; (3) sufficient long propagation length beyond hundreds of micrometers or even millimeters. To relieve the first obstacle, employing multilayer graphene structures can efficiently control the field localizations and effective refractive index by varying the number of the graphene layers, thus overcoming the difficulty of exciting the high-*k* SPP modes. Next, the coupling efficiency can be improved by designing perfectly adiabatic metallic gratings, reducing field scattering during the coupling process. The final one is a common limit, as the SPP modes supported by general noble metals operating in the near-IR and visible light bands. In addition to discovering new low-loss materials, designing a novel waveguide structure by combining multiple guiding mechanisms together has been the most effective solution to decrease the propagation losses.

## 4. Conclusions

This work reported a mid-IR waveguiding structure based on MLGMTs on a Si nano-rib waveguide structure covered by a porous SiO_2_ layer. By coupling the low-loss fundamental VPP mode of the MLGMTs, which is formed by coupling the SPP modes at individual graphene sheets and the dielectric mode of a Si nano-rib, the hybrid mode of the present design achieves an ultralong propagation length *L_p_* = 19.4 µm with *A_m_* = 2.6 × 10^−6^ at *E_F_* = 0.6 eV operating at *f* = 30 THz. Compared with the reported results, the *L_p_* of our structure is five times greater than those reported at a comparable *A_m_*. Even for the looser *A_m_*, previously reported *L_p_* values at *E_F_* = 0.6 eV and *f* = 30 THz were still limited to below 10 µm. The MLGMTs provide a high-conductivity graphene structure that significantly increases *L_p_* with an increasing number of graphene layers. Therefore, the increased degree of *L_p_* is mainly restricted by the modern fabrication technique. In addition, the crosstalk between two adjacent waveguides demonstrates that the proposed structure is beneficial in realizing high-integration photonic devices operating in the mid-IR band. Our design is expected to pave the way for potential applications in building ultralow loss and compact and tunable mid-IR photonic devices and can be extended to other extraordinary 2D materials.

## Figures and Tables

**Table 1:** Comparisons of the modal properties of *A_m_*, *L_p_*, and *FoM*.

Reference	*A_m_*	*L_p_* (µm)	*FoM*	*E_F_* (eV)
[32]	8.0 × 10^−7^	5	4951	0.8
[34]	9.0 × 10^−4^	4	118	0.6
[36]	9.0 × 10^−5^	4	374	0.6
[38]	1.0 × 10^−5^	10	2803	0.6
[39]	2.0 × 10^−6^	3.5	2193	0.6
This work	2.6 × 10^−6^	19.4	10,612	0.6

## Data Availability

The data presented in this study are available on request from the corresponding author.

## References

[1] Barnes W.L., Dereux A., Ebbesen T.W. (2003). Surface plasmon subwavelength optics. Nature.

[2] Gramotnev D.K., Bozhevolnyi S.I. (2010). Plasmonics beyond the diffraction limit. Nat. Photonics.

[3] Han Z., Bozhevolnyi S.I. (2013). Radiation guiding with surface plasmon polaritons. Rep. Prog. Phys..

[4] Bian Y., Gong Q. (2014). Highly confined guiding of low-loss plasmon waves in hybrid metal-dielectric slot waveguides. Nanotechnology.

[5] Bian Y., Ren Q., Kang L., Yue T., Werner P.L., Werner D.H. (2018). Deep-subwavelength light transmission in hybrid nanowire-loaded silicon nano-rib waveguides. Photonics Res..

[6] Stanley R. (2012). Plasmonics in the mid-infrared. Nat. Photonics.

[7] Zhong Y., Malagari S.D., Hamilton T., Wasserman D.M. (2015). Review of mid-infrared plasmonic materials. J. Nanophotonics.

[8] Grigorenko A.N., Polini M., Novoselov K.S. (2012). Graphene plasmonics. Nat. Photonics.

[9] Low T., Avouris P. (2014). Graphene plasmonic for terahertz to mid-infrared applications. ACS Nano.

[10] Politano P., Chiarello G. (2014). Plasmon modes in graphene: Status and prospect. Nanoscale.

[11] Gon P.A.D., Peres N.M.R. (2016). An Introduction to Graphene Plasmonics.

[12] Li Y., Tantiwanichapan K., Swan A.K., Paiella R. (2020). Graphene plasmonic devices for terahertz optoelectronics. Nanophotonics.

[13] Bao Q., Zhang H., Wang B., Ni Z., Haley C., Lim Y.X., Wang Y., Tang D.Y., Loh K.P. (2011). Broadband graphene polarizer. Nat. Commun..

[14] Pei C., Yang L., Wang G., Wang Y., Jiang X., Hao Y., Li Y., Yang J. (2015). Broadband graphene/glass hybrid waveguide polarizer. IEEE Photonics Technol. Lett..

[15] He X., Liu J. (2017). Flexible and broadband graphene polarizer based on surface silicon-core microfiber. Opt. Mater. Express.

[16] Kim J.T., Choi H. (2018). Polarization control in graphene-based polymer waveguide polarizer. Laser Photonics Rev..

[17] Liu M., Yin X., Ulin-Avila E., Geng B., Zentgraf T., Ju L., Wang F., Zhang X. (2011). A graphene-based broadband optical modulator. Nature.

[18] Youngblood N., Anugrah Y., Ma R., Koester S.K., Li M. (2014). Multifunctional graphene optical modulator and photodetector integrated on silicon waveguides. Nano Lett..

[19] Ansell D., Radko I.P., Han Z., Rodriguez F.J., Bozhevolnyi S.I., Grigorenko A.N. (2015). Hybrid graphene plasmonic waveguide modulators. Nat. Commun..

[20] Hao R., Jiao J., Peng X., Zhen Z., Dagarbek R., Zou Y., Li E. (2019). Experimental demonstration of a graphene-based hybrid plasmonic modulator. Opt. Lett..

[21] Ding Y., Guan X., Zhu X., Hu H., Bozhevolnyi S.I., Oxenløwe L.K., Jin K.J., Mortensen N.A., Xiao S. (2017). Efficient electro-optic modulation in low-loss graphene-plasmonic slot waveguides. Nanoscale.

[22] Yuana W., Shi G. (2013). Graphene-based gas sensors. J. Mater. Chem. A.

[23] Peters A., Turvey S., Horsfall A.B. (2019). High-temperature Hall effect sensor based on epitaxial graphene on high-purity semiinsulating 4H-SiC. IEEE Trans. Electron Dev..

[24] Choi J.H., Lee J., Byeon M., Hong T.E., Park H., Lee C.Y. (2020). Graphene-based gas sensors with high sensitivity and minimal sensor-to-sensor variation. ACS Appl. Nano Mater..

[25] Ghorbanzadeh M., Darbari S., Moravvej-Farshia M.K. (2016). Graphene-based plasmonic force switch. Appl. Phys. Lett..

[26] Dolleman R.J., Belardinelli P., Houri S., van der Zant H.S.J., Alijani F., Steeneken P.G. (2019). High-frequency stochastic switching of graphene resonators near room temperature. Nano Lett..

[27] Cox J.D., de Abajo F.J.G. (2019). Single-plasmon thermo-optical switching in graphene. Nano Lett..

[28] Ono M., Hata M., Tsunekawa M., Nozaki K., Sumikura H., Chiba H., Notomi M. (2020). Ultrafast and energy-efficient all-optical switching with graphene-loaded deep-subwavelength plasmonic waveguides. Nat. Photonics.

[29] Ye L., Sui K., Feng H. (2020). High-efficiency couplers for graphene surface plasmon polaritons in the mid-infrared region. Opt. Lett..

[30] Sun Y., Bian Y., Zhao X., Zheng Z., Liu J., Liu J. Low-loss graphene plasmonic waveguide based on a high-index dielectric wedge for tight optical confinement. Proceedings of the OSA Technical Digest (Optical Society of America, 2013).

[31] Xu W., Zhu Z.H., Liu K., Zhang J.F., Yuan X.D., Lu Q.S., Qin S.Q. (2015). Dielectric loaded graphene plasmon waveguide. Opt. Express.

[32] Liu J.P., Zhai X., Wang L.L., Li H.J., Xie F., Xia S.X., Shang X.J., Luo X. (2016). Graphene-based long-range SPP hybrid waveguide with ultra-long propagation length in mid-infrared range. Opt. Express.

[33] Bahadori-Haghighi S., Ghayour R., Sheikhi M.H. (2018). Design and analysis of low loss plasmonic waveguide and directional coupler based on pattern-free suspended graphene sheets. Carbon.

[34] Gao Y., Ren G., Zhu B., Wang J., Jian S. (2014). Single-mode graphene-coated nanowire plasmonic waveguide. Opt. Lett..

[35] Gao Y., Ren G., Zhu B., Liu H., Lian Y., Jian S. (2014). Analytical model for plasmon modes in graphene-coated nanowire. Opt. Express.

[36] Teng D., Wang K., Li Z., Zhao Y. (2019). Graphene-coated nanowire dimers for deep subwavelength waveguiding in mid-infrared range. Opt. Express.

[37] Liu J.P., Zhai X., Wang L.L., Li H.J., Xie F., Lin Q., Xia S.X. (2016). Analysis of mid-infrared surface plasmon modes in a graphene-based cylindrical hybrid waveguide. Plasmonics.

[38] Liu J.P., Zhai X., Xie F., Wang L.L., Xia S.X., Li H.J., Luo X., Shang X.J. (2017). Analytical model of mid-infrared surface plasmon modes in a cylindrical long-range waveguide with double-layer graphene. J. Lightwave Technol..

[39] Teng D., Wang K., Huan Q., Chen W., Li Z. (2020). High-performance light transmission based on graphene plasmonic waveguides. J. Mater. Chem. C.

[40] Oulton R.F., Sorger V.J., Genov D., Pile D., Zhang X. (2008). A hybrid plasmonic waveguide for subwavelength confinement and long-range propagation. Nat. Photonics.

[41] Chen B., Meng C., Yang Z., Li W., Lin S., Gu T., Guo X., Wang D., Yu S., Wong C.W. (2014). Graphene coated ZnO nanowire optical waveguides. Opt. Express.

[42] Cao T., Tian L., Liang H., Qin K.R. (2018). Reconfigurable, graphene-coated, chalcogenide nanowires with a sub-10-nm enantioselective sorting capability. Microsyst. Nanoeng..

[43] Iorsh I.V., Mukhin I.S., Shadrivov I.V., Belov P.A., Kivshar Y.S. (2013). Hyperbolic metamaterials based on multilayer graphene structures. Phys. Rev. B.

[44] Othman M.A.K., Guclu C., Capolino F. (2013). Graphene-based tunable hyperbolic metamaterials and enhanced near-field absorption. Opt. Express.

[45] Smirnova D.A., Iorsh I.V., Shadrivov I.V., Kivshar Y.S. (2014). Multilayer graphene waveguides. JETH Lett..

[46] Schubert E.F., Kim J.K., Xi J.Q. (2007). Low-refractive-index materials: A new class of optical thin-film materials. Phys. Status Solidi B.

[47] Lin J.Y., Liu W., Smart J.A. (2007). Optical thin-film materials with low refractive index for broadband elimination of Fresnel reflection. Nat. Photonics.

[48] Yan H., Li X., Chandra B., Tulevski G., Wu Y., Freitag M., Zhu W., Avouris P., Xia F. (2012). Tunable infrared plasmonic devices using graphene/insulator stacks. Nat. Nanotechnol..

[49] Chang Y.C., Liu C.H., Liu C.H., Zhang S., Marder S.R., Narimanov E.E., Zhong Z., Norris T.B. (2015). Realization of mid-infrared graphene hyperbolic metamaterials. Nat. Commun..

[50] Baitimirova M., Viter R., Andzane J., Lee A., Voiry D., Iatsunskyi I., Coy E., Mikoliunaite L., Tumenas S., Załęski K. (2016). Tuning of structural and optical properties of graphene/ZnO nanolaminates. J. Phys. Chem. C.

[51] Yang Y., Lin H., Zhang B.Y., Zhang Y., Zheng X., Yu A., Hong M., Jia B. (2019). Graphene-based multilayered metamaterials with phototunable architecture for on-chip photonic devices. ACS Photonics.

[52] Lin H., Sturmberg B.C.P., Lin K., Yang Y., Zheng X., Chong T.K., Martijn de Sterke C., Jia B. (2019). A 90-nm-thick graphene metamaterial for strong and extremely broadband absorption of unpolarized light. Nat. Photonics.

[53] Lee C.C., Suzuki S., Xie W., Schibli T.R. (2012). Broadband graphene electro-optic modulators with sub-wavelength thickness. Opt. Express.

[54] Fan M., Yang H., Zheng P., Hu G., Yun B., Cui Y. (2017). Multilayer graphene electro-absorption optical modulator based on double-stripe silicon nitride waveguide. Opt. Express.

[55] Sun D., Divin C., Berger C., De Heer W.A., First P.N., Norris T.B. (2010). Spectroscopic measurement of interlayer screening in multilayer epitaxial graphene. Phys. Rev. Lett..

[56] Bass M., DeCusatis C., Enoch J., Lakshminarayanan V., Li G., MacDonald C., Mahajan V., StrylandBass E.V. (2009). Handbook of Optics, Third Edition Volume IV: Optical Properties of Materials, Nonlinear Optics, Quantum Optics.

[57] Falkovsky L.A., Pershoguba S.S. (2007). Optical far-infrared properties of a graphene monolayer and multilayer. Phys. Rev. B.

[58] Huang W.P. (1994). Coupled-mode theory for optical waveguides: An overview. J. Opt. Soc. Am. A.

[59] Veronis G., Fan S.H. (2008). Crosstalk between three-dimensional plasmonic slot waveguides. Opt. Express.

